# Supplementary material for: Cost and Effectiveness of Long-Term Care Following Integrated Discharge Planning: A Prospective Cohort Study
Source: Healthcare (Basel). 2021 Oct 21;9(11):1413. doi: 10.3390/healthcare9111413 (PMC8621918; doi:10.3390/healthcare9111413)
Supplement: Supplementary file 1 [file healthcare-09-01413-s001.zip › healthcare-1384856-supplementary.pdf]

Supplementary Table S1. Comparison of patient characteristics between with follow-up and without follow-up in the standard transition cohort *

Variables		Follow-Up Group (n = 49)	Without Follow-Up Group (n = 135)	p Value §
Demographic characteristics				
Age, years		80.88 ± 9.96	77.56 ± 12.35	0.092
Gender	Male	33(67.3%)	88(65.2%)	0.922
	Female	16(32.7%)	47(34.8%)	
Body mass index, km/m^2		22.31 ± 4.24	23.01 ± 4.43	0.345
Education, years		7.88 ± 5.37	6.99 ± 4.75	0.282
Marital status	Single	5(10.2%)	12(8.9%)	0.963
	Married	32(65.3%)	89(65.9%)	
	Widowed	12(24.5%)	34(25.2%)	
Family support †		1.08 ± 0.53	0.96 ± 0.50	0.140
Smoking	Yes	3(6.1%)	14(10.4%)	0.566
Drinking	Yes	5(10.2%)	16(11.9%)	0.961
Clinical characteristics				
Charlson Comorbidity Index, score		4.25 ± 2.64	4.59 ± 3.29	0.515
Multimorbidity Frailty Index		0.15 ± 0.87	0.15 ± 0.11	0.898
Activities of Daily Living †	0	3(6.1%)	29(21.5%)	0.057
	1	8(16.3%)	13(9.6%)	
	2	20(40.8%)	41(30.4%)	
	3	18(36.7%)	52(38.5%)	
Malnutrition Universal Screening Tool †	0	33(67.3%)	89(65.9%)	0.763
	1	4(8.2%)	16(11.9%)	
	2	12(24.5%)	30(22.2%)	
Discharged with urinary catheter	Yes	10(20.4%)	22(16.3%)	0.667

* Values are mean ± standard deviation or n (%). § Independent t test or Chi square tests p value. † Family support: living or stay with spouse (yes 1, no 0), living with parents (yes 1, no 0), children (yes 1, no 0); activities of daily living: 0 completely independent, 1 mild disability, 2 moderate disability, 3 severe disability; Malnutrition Universal Screening Tool: 0 low risk, 1 mild risk, 2 high risk.
